# Mapping Environmental Dimensions of Dengue Fever Transmission Risk in the Aburrá Valley, Colombia

**DOI:** 10.3390/ijerph6123040

**Published:** 2009-12-02

**Authors:** Sair Arboleda, Jaramillo-O. Nicolas, A. Townsend Peterson

**Affiliations:** 1 Grupo de Biología y Control de Enfermedades Infecciosas (BCEI), Instituto de Biología, Universidad de Antioquia, Sede de Investigaciones Universitarias, SIU, Calle 62 # 52–59 Laboratory 620, P.O. Box: 1226, Medellín, Colombia; E-Mails: sairorieta@yahoo.es (S.A.); nicolas.jaramillo@siu.udea.edu.co (N.J-O.); 2 Natural History Museum, the University of Kansas, Lawrence, KS 66045, USA

**Keywords:** dengue fever, remote sensing, geographic information systems, ecological niche modeling

## Abstract

Dengue fever (DF) is endemic in Medellín, the second largest Colombian city, and surrounding municipalities. We used DF case and satellite environmental data to investigate conditions associated with suitable areas for DF occurrence in 2008 in three municipalities (Bello, Medellín and Itagüí). We develop spatially stratified tests of ecological niche models, and found generally good predictive ability, with all model tests yielding results significantly better than random expectations. We concluded that Bello and Medellín present ecological conditions somewhat different from, and more suitable for DF than, those of Itagüí. We suggest that areas predicted by our models as suitable for DF could be considered as at-risk, and could be used to guide campaigns for DF prevention in these municipalities.

## 1. Introduction

Dengue fever (DF) is an arboviral disease transmitted to humans by mosquitoes of the genus *Aedes* [1]; its transmission is determined by factors including mosquito density, circulating virus serotypes, and susceptibility of human populations [2]. Since no vaccine or specific treatment is available, the only prevention for dengue is vector control; this situation places a premium on identification and prediction of risk areas as best means of dengue prevention. Some previous studies have used geographic information systems (GIS) to develop such hypotheses [3–13] under a regional-scale perspective, few extrapolate results across broad areas to test the predictive ability of the “model” proposed [14].

Transmission dynamics of vector-borne diseases are inherently spatial processes, so variables linked to vector or case distributions may be a useful basis on which to predict spatial dimensions of transmission in unsampled areas [15]. Occurrence of DF is determined by multiple factors, including environmental dimensions that affect the population biology, development, and behavior of vectors, as well as dimensions that determine the population biology and natural history of the viruses, and even the behavior of humans. Across continental extents and broad areas, environmental factors like humidity, temperature, and rainfall are known determinants of dengue vector development that can limit DF occurrence [16–18]. As such, when mosquito occurrence data are not available, DF case data provide a useful basis available for estimating occurrence patterns [19]; indeed, Ostfeld *et al.* [15] argued that risk maps based on case-occurrence data may be optimal, as they incorporate all risk factors in a single view.

Effective risk assessment, however, requires a broader-scale and predictive point of view: assessing risk cannot be confined to areas already sampled, but rather should be extendable to novel, unsampled areas in some predictive fashion. One approach to achieving such a goal is by modeling species’ ecological and environmental requirements, which can be termed ecological niche modeling (ENM). In ENM, known occurrences of species are related to raster (grid format) geospatial datasets describing aspects of the environmental landscape, to derive a quantitative model of the ecological niche (defined as the suite of conditions under which the species can maintain populations without immigrational input). This niche model is then tested for significant predictive ability, and can be projected onto landscapes to estimate a potential geographic distributional area for the species.

Here, we take a municipality-scale approach to risk assessment for DF: we associate DF cases in 2008 across the Aburrá Valley (Antioquia, Colombia) with environmental factors describing aspects of surface reflectance and topography. We develop and test predictive spatial models of DF occurrence for three municipalities where the mosquito *Aedes aegypti* is the only dengue vector known, testing the degree to which models developed in one area can be used to anticipate patterns of DF occurrence in other areas. Hence, this paper describes an exploration of ecological niche dimensions and associated geographic distributions for DF cases.

## 2. Materials and Methods

### 2.1. Input Data

#### Dengue fever cases.

**Figure 1.**
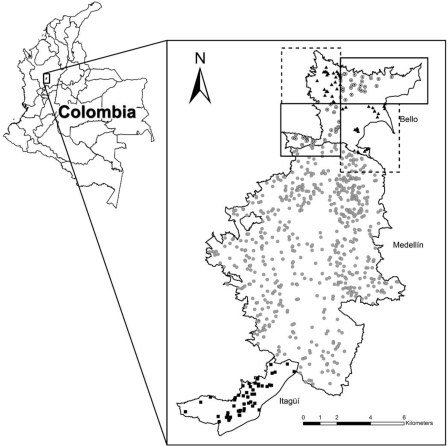
Map of Colombia showing Bello, Medellín, and Itagüí, with the occurrence data for dengue fever used in the study. The Bello municipality data are shown divided in quadrants: northwestern-southeastern (triangles in quadrants with dashed line) and northeastern-southwestern (circles in quadrants with continuous line).

Symptomatic reported DF case occurrence data were obtained for 2008 from municipal health departments in Bello, Medellín, and Itagüí (Antioquia, Colombia); the first two municipalities are considered endemic for DF, whereas the latter shows only sporadic cases. Symptoms considered as indicative of probable DF included acute illness with two or more of the following manifestations: headache, retro-orbital pain, myalgia, arthralgia, rash, hemorrhagic manifestations, leucopenia; and supportive serology (*i.e.*, a reciprocal haemagglutination-inhibition antibody titre ≥ 1,280, a comparable IgG enzyme-linked immunosorbent assay (ELISA) titre, or a positive IgM antibody test on a late-acute or convalescent-phase serum specimen); or occurrence at the same location and time as other confirmed cases of dengue fever [20]. Case-occurrence data were provided to us at the patient address level: of the total of 1,169 DF cases reported, 113 localities for Bello, 611 for Medellín, and 52 for Itagüí could be geo-referenced with a spatial precision of 20 m or finer (Figure 1).

Given the fact that the home address is not necessarily the infection site, and the occurrence data thus include certain amounts of error, we considered a base level of omission error that is expected owing to these complications, which has been quantified as parameter *E* [21]; we selected *E* = 10 as an appropriate level: that is, we expected occurrence data to include as much as 10% error, given problems with geo-referencing and with identification of true exposure sites.

#### Environmental data.

We sought fine-resolution geospatial data characterizing environmental variation across Colombian landscapes for the time period of interest. We estimate potential risk areas for dengue case occurrences based on indirect, landscape-scale measures that are correlates of environmental factors associated with the disease-vector-host interactions that make up the etiology of this disease. In particular, we focus on correlates of two key environmental dimensions: temperature and vegetation; the Landsat imagery we use are estimators of these variables (Table 1) [22]; spatial resolution 30 m; Table 1) For reasons of simplicity, we limited our analyses to the seven raw data “bands” plus one vegetation index, as ongoing experimentation has suggested that addition of other vegetation indices do not add dramatically to the predictive power of ecological niche models [23].

In sum, the environmental data set included 11 digital data layers summarizing aspects of topography (elevation, aspect, and slope) from the Shuttle Radar Topography Mission (SRTM) sensor (native spatial resolution 90 m), and remotely-sensed data layers from 1 February, 5 April, and 12 September 2008, for 7 spectral bands and the Normalized Difference Vegetation Index (NDVI; see band descriptions in Table 1) from the Landsat 7 satellite’s Enhanced Thematic Mapper plus (ETM+) sensor (native spatial resolution 30 m; path/row 9/56, UTM projection, USGS provider: http://glovis.usgs.gov/). NDVI is a numerical quantity derived from reflectance measured in the red and near-infrared spectral bands that provides information about photosynthetic activity [24]. All layers were resampled to 30 m resolution for analysis, and each individual Landsat image was used as an environmental predictor in model development. Although this suite of environmental data layers may not provide variables that are immediately and easily interpretable (e.g., relative humidity, precipitation), this choice on our part was deliberate: we maximized the quality of our predictions, but at the cost of the ease of environmental interpretation of the models.

### 2.2. The Maxent Algorithm

We used the Maxent algorithm for modeling the ecological niche and spatial distribution of DF cases [25]. Maxent is a general-purpose algorithm that generates predictions from incomplete sets of information based on a probabilistic framework. As far as the details of its inferential approach, Maxent relies on the assumption that the incomplete empirical probability distribution (which is based on the species’ occurrences) can be approximated with a probability distribution of maximum entropy, subject to certain environmental constraints, and that this distribution approximates a species’ potential ecological distribution [25]. Like most maximum likelihood estimation approaches, Maxent, assumes *a priori* a uniform distribution, and performs a series of iterations in which weights are adjusted to maximize the average probability of the point localities, expressed as the training gain [25]. Within the processing of the Maxent program, these weights are then used to compute the maximum entropy probability distribution over the entire geographic space, with values expressing the environmental suitability of each grid cell as a function of the environmental conditions presented there. A high value of the function in a particular grid cell indicates suitable conditions for that species [25], or in this case for DF case-occurrences. We used Maxent version 3.3.0-beta, with logistic output and a random test percentage of 50%; all other settings were default.

Predictive models of disease occurrence may be good or bad, but model quality can be ascertained only via evaluation with independent testing data, preferably which are spatially independent of the training data to avoid problems caused by spatial autocorrelation and nonindependence of points [26]. Because only data documenting *presence* of DF cases were available for this study (*i.e.*, no data were available to document that DF was absent at particular sites), we used a binomial probability approach to model validation: we compared observed model performance to that expected under a null hypothesis of random association between model predictions and test point distribution. Because such tests require binary (*i.e.*, yes-no) predictions, our first step was to convert raw (continuous) predictions to binary predictions via a modification of the least training presence threshold approach of Pearson *et al.* [27]: we choose as a threshold value the suitability score that included 100-*E* percent (as opposed to 100%) of training points, and thus took into account the error believed to be inherent in the input data [21]. In the binomial test, the number of test points was taken as the number of trials, the number of correctly predicted test points as the number of successes, and the proportion of the study area predicted present as the probability of a success if predictions and points were associated at random [28]. All testing was carried out in a series of spatially stratified tests, as is detailed below. These tests thus evaluated the ability of models to anticipate DF case distributions across unsampled areas, considering a model as validated if it predicts case distributions better than a “model” making random predictions, and as such these tests are considerably more stringent than simple random partitions of occurrence data or cross-validation exercises.

### 2.3. Model Testing Approach

We carried out a series of tests within one municipality and among municipalities. In each case, in a first step, *within the training region*, we evaluated model predictivity in a series of test of models based on different combinations of ecological dimensions to identify optimal combinations of variables on that particular landscape. We trained models with all variables (*N* = 11), and then with each combination of *N*-1 variables, with each variable alone, with the Landsat data only, and with the topographic variables only. The model prediction was then converted to binary and a cumulative binomial probability calculated as described above; we then chose the best combination of environmental coverages for inter-regional predictions based on these probabilities. We note that all of these tests were conducted within the training region, and thus do not affect the independent nature of our spatially stratified tests. Hence, in all, each model was tested with 24 variable combinations.

#### Predictivity within a municipality.

We analyzed predictivity between DF cases in two sectors of Bello by dividing the municipality into quadrants based on the median latitude and median longitude of DF cases in 2008. We used northeast-southwest and northwest-southeast quadrants for training and testing models (Figure 1), respectively, and then switched the roles for another set of tests. This sub-setting strategy was used to test the null hypothesis that ENMs would be unable to predict into unsampled areas. Hence, two tests (the two reciprocal predictions) were carried out within Bello.

#### Predictivity between municipalities.

We used occurrence data from each pair of municipalities to train ENMs, which we projected onto the third municipality; DF cases in the third municipality were used to test model predictivity. Given the availability of occurrence data for Bello, Medellín, and Itagüí in 2008, three tests were carried out: Bello and Medellín predicting Itagüí, Bello and Itagüí predicting Medellín, and Medellín and Itagüí predicting Bello.

Finally, we wished to develop a single overall model that represents the best-available picture of DF case-occurrence risk across the region, albeit not including statistical testing as above. This model was built using all occurrence data available, based on Landsat variables and slope. To assess uncertainty in predictions based on all DF case-occurrence information, we built 100 models each based on a random 50% of the occurrence data chosen at random without replacement. These models thus capture the degree to which DF case occurrence data availability may drive the results of the analyses, and we consider areas that are predicted consistently in all of these replicate analyses as most certain. Hence, the degree to which sum of these replicate maps (each thresholded as described above) was less than 100 as used to represent uncertainty in the overall risk map.

### 2.4. Niche Characterization

To explore environmental factors associated with positive and negative predictions of suitability for DF transmission, we explored further the model based in all points. We plotted 10,000 points randomly across areas of the municipalities predicted as absent or present by this model. We then assigned the value of each input environmental and topographic layer to each of the random points, and exported the associated attributes table in ASCII format. This table was used for comparisons of environmental characteristics of areas predicted as suitable and unsuitable.

## 3. Results

Multiple comparisons conducted in this study showed that coincidence between spatial predictions and independent test points was significantly better than random expectations, suggesting in general that ENMs had predictive power regarding DF case distributions (Table 2). That is, models based on known human DF cases can help to anticipate spatial distributions of DF cases in other areas better than random expectations.

### 3.1. Predictivity within a Municipality

**Figure 2.**
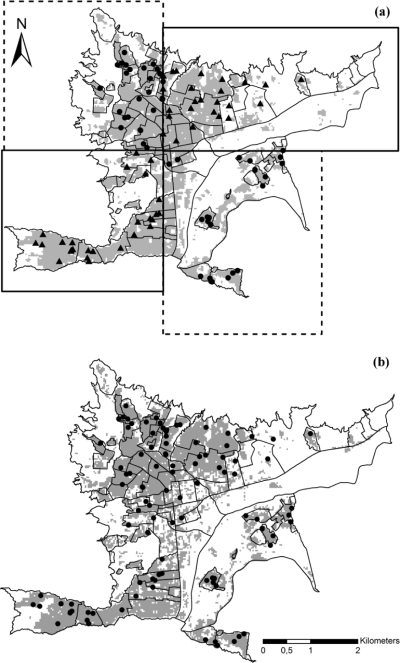
Predicted areas for dengue fever cases within Bello. (a) Predicted area using the subset of occurrence data in the northwestern-southeastern area; dots refer to training cases and triangles to testing cases. (b) Predicted area using the whole dataset of Bello dengue fever cases; dots indicate the cases reported in Bello in 2008. Gray areas are those predicted as suitable for dengue fever cases.

The two tests based within Bello showed significant predictive power of the ENMs (both *P* << 0.05; Table 2). When the model was evaluated with respect to different ecological dimensions, aspect, slope, and band 6 were not informative regarding DF case distributions, so these variables were excluded from analysis (Table 2). Figure 2 shows the model based on the remaining variables for one of the spatial subsets of data points (Figure 2a), as well as the model based on the entire Bello dataset (Figure 2b). In both models, areas identified as suitable for DF cases coincided with urbanized areas, and >89% of DF cases fell into predicted areas (Table 2, Figure 2). The model based on the whole Bello dataset predicted a more restricted area as suitable. In summary, spatially stratified tests within Bello indicated significant ENM predictivity of DF case occurrences, even when predicting into unsampled areas.

### 3.2. Predicting between Municipalities

**Figure 3.**
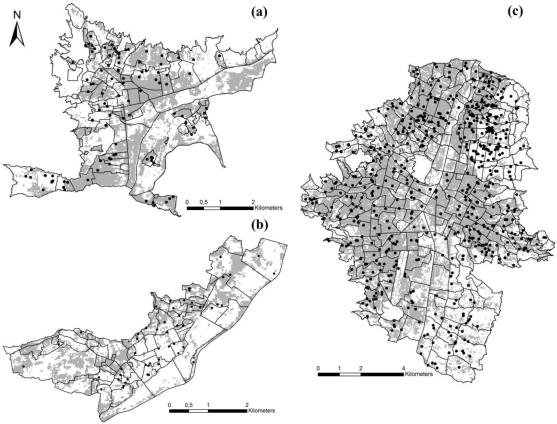
Predicted area for dengue fever cases in Bello, Medellín, and Itagüí, each developed based on independent testing data from the remaining two municipalities. Occurrences shown in each municipality are thus independent testing data. Gray areas are those predicted as suitable for dengue fever cases.

In the three between-municipality tests (Table 2, Figure 3), all models showed predictive ability statistically significantly better than random expectations, and DF cases coincided in great percentage with predicted areas (Table 2, Figure 3). Because we optimized variable inclusion for each model, variable sets on which models were based differed between tests. Hence, when DF cases from Medellín and Itagüí were used as training data for predictions in Bello, we excluded aspect and slope (Table 2); here, 64% of the data points fell into predicted area (Figure 3a), which was more restricted (45% of the area of the municipality) than the area predicted with DF data from within Bello. It is intriguing that, despite the fact that this prediction for Bello was made with data from the other municipalities, the area predicted is very similar to that based on DF cases from within Bello. The model trained with DF cases from Bello and Medellín and projected to Itagüí was built with aspect and elevation excluded (Figure 3b); suitable areas were concentrated in the western part of the region, and the predicted area (37%) omitted some DF occurrence points; we note that model projections to Itagüí were particularly unstable in terms of proportional area predicted as suitable. Still, the prediction, which included 56% of testing points, is better than random expectations (*P* < 0.05; Table 2). Finally, the model built for Medellín using Bello and Itagüí DF cases was optimized when based on Landsat variables only. In Medellín, 55% of the area was identified as DF-suitable, concentrated in the western and central-eastern parts of the region; some areas in eastern Medellín were predicted as unsuitable (*i.e.*, low risk) for DF occurrence (correctly predicting 68% of testing points; Figure 3c).

### 3.3. Predicting DF Cases in Aburrá Valley

**Figure 4.**
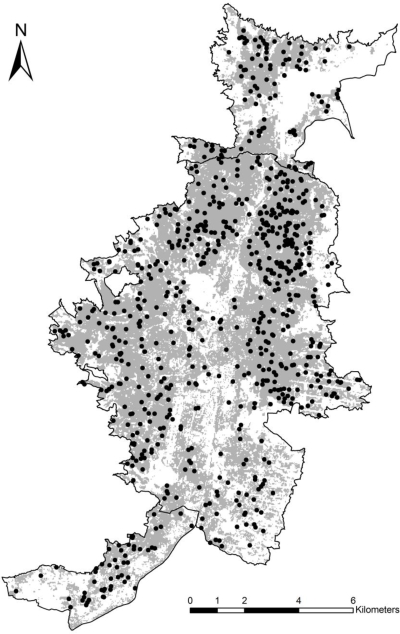
Final risk model for dengue fever case-occurrences in three municipalities of Aburrá Valley. Gray areas are areas predicted as suitable for DF cases; black points are the 2008 cases of dengue fever.

The model was evaluated with respect to different ecological dimensions, and the best model (Figure 4) excluded aspect. Comparing between areas predicted within each municipality using the data from that municipality with the predictions based on DF case-occurrences from all three municipalities, we found that the latter predicted broader areas, at times reducing omission errors (Table 2). Hence, our initial exercises of prediction within and between municipalities were useful in assessing the degree to which predictivity into unsampled areas could be achieved; however, the entire dataset offered a more comprehensive overview of DF transmission across the region.

### 3.4. Niche Characteristics

**Figure 5.**
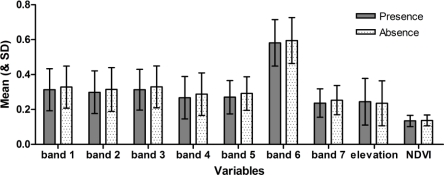
Bar graph showing means and standard deviations for each environmental variable in areas predicted as suitable and unsuitable for dengue fever cases across the study area.

Variable-by-variable comparisons of areas predicted as suitable versus unsuitable in the model based on all DF case data showed no significant univariate differences (Figure 5). In fact, all environmental variables showed only subtle differences between suitable and unsuitable areas. Hence, in discriminating between suitable and unsuitable areas, no single variable offers clear separation. Rather, differentiation in suitability is clearly in multivariate space, and depends on complex combinations of variables.

## 4. Discussion

Our work is based on cases diagnosed based on clinical symptoms, but relatively few confirmed cases (about 10%); we are well aware of the disadvantages of such data sets; but reliance on the relatively few laboratory confirmed cases would bring its own biases, particularly as regards paucity of information. It is true that DF diagnosis is difficult, that apparent DF cases can be confused with other diseases (even down to a simple case of the flu), and that a certain proportion of cases occur without clinical symptoms, which may lead to erroneous associations of environmental conditions with DF transmission. Another source of error is the place of exposure, because people do not necessarily contract DF at their residences (*i.e*., the address reported); rather, they may get infected at work or at school. Sometimes, actual infection sites are not even located in the same municipality, and hence may confuse the model still further. These uncertainties in our basic data sets introduce what can be considered a ‘basement’ level of error, limiting the maximum possible predictive ability of models, as has been noted previously in similar analyses of leishmaniasis cases in southeastern Brazil [29]. We have attempted to take into account the effects of these known error sources using the *E* parameter [21], but their effects may be manifested in the less stable results for predictions in Itagüí. More generally, the use of *E* offers a quantitative means of incorporating known inherent error levels in spatial analyses, with the aim of not overinterpreting data, when the data are known to have noise along with the signal [21].

In general, the variables excluded in all models (Table 2) were aspect, slope, elevation, and Landsat band 6. The first two of these variables are likely not important because breeding sites for *Ae. aegypti* are artificial containers, rather than broader-scale features that might associate with standing water. Elevation varies quite dramatically across the valley, and so is likely also not associated importantly with presence *versus* absence of dengue cases. Finally, band 6 reflects temperature, which again may be at best only indirectly associated with dengue transmission. However, we emphasize that models serve two distinct purposes—prediction and explanation—and that ours were distinctly purposed from the outset of the study towards the former, and not so much towards the latter.

Our tests that used spatial subsetting of DF cases within the municipality of Bello showed similar spatial patterns between the two reciprocal models. The significant predictions in each case, plus this similarity, allowed us to reject the null hypothesis of no association between predictions and independent test points—this significant result indicates that the models indeed have significant predictive ability. Hence, across extents of 5–10 kilometers, our ENM-based results were able to predict DF transmission potential even unsampled areas, at least at this spatial extent. Similar work by Siqueira-Junior *et al*. [12] also concluded that case occurrence data could provide a useful basis for guiding control interventions.

At broader extents (*i.e.*, over an area of 100 km^2^), our ENMs were able to project DF transmission risk among municipalities. We were able to validate these predictions by means of testing with independent data from each municipality (Table 2, Figure 3). It is interesting to see that the predictions within Bello and Medellín (both DF-endemic) showed broad areas predicted as at risk, while Itagüí showed smaller areas as at risk, which may be concordant with its non-endemic status. Because the three municipalities have similar policies regarding dengue prevention and control, the differences detected are probably a function of environmental characteristics and consequent suitability for DF transmission, rather than of differences in immunological status of the human populations. Analyzing all of the DF case-occurrence data from the three municipalities of the Aburrá Valley, we found that the areas predicted as suitable were broader than the areas predicted by the models for individual municipalities—these differences probably result from the broader environmental diversity of the overall area, which is not considered in subsampled models. We note, however, that our explorations of niche characteristics as estimated in our models suggest that no single environmental dimension dominates in delimiting the DF niche; rather, the niche is a complex amalgam of all of the factors included in each model.

The coarse-resolution view of our results is that factors determining DF case occurrences are well-known: high humidity and temperature. At finer resolutions, however, additional factors can enter into the picture. These factors range from human social dimensions to microclimatic and substrate-based variables. In comparing Itagüí with the remaining municipalities, its intrinsic physical characteristics make it different from the other two studied herein, at least from the standpoint of conditions modeled as appropriate for DF case occurrences. This result is important, as it leads us to revisit the scale at which this sort of analysis is normally carried out (e.g., within a municipality), suggesting that patterns documented in one area may not map conveniently onto other areas where we may not have data with which to validate predictions.

Mapping disease transmission risk is, of course, a multi-dimensional task. That is, disease transmission (in this case for DF) depends on vector distribution and biology, pathogen population dynamics, characteristics of the host population (in the DF case, infected humans), and characteristics of the at-risk populations [30]. In the present case, however, we have focused on overall DF case distributions in one year, and have not as yet built more parsed maps based on individual factors—rather, we use the ‘black box’ of DF case occurrence distributions to train models. We also hope to develop inter-year comparisons at some point, depending on access to the appropriate data, as this step would allow assessment of “good” versus “bad” periods for DF and how they relate to environmental variation. This approach has the advantage of subsuming the multitude of causal factors into a single quantity [15]; however, it does not provide the full detail of where vectors are present, where humans are present, *etc*.—such detail would be desirable in a fully developed risk assessment. Niche models such as we have developed, in tandem with spatial information on environmental risk factors [31] and entomological indices [32–34], could help focus surveillance and control efforts still more. As such, we suggest that further exploration of such risk-mapping approaches for DF and other diseases may prove rewarding.

Finally, we compare these approaches based on distributions of species or disease transmission events in environmental spaces with the more traditional ‘landscape epidemiology’ approaches that are based on surfaces fit in geographic dimensions only (e.g., [35]). These geographic-space risk assessments are able only to interpolate existing patterns among known data, but no means of extrapolation to other areas are available. The environment-based approaches explored here, in contrast, build the ‘model’ in environmental dimensions, and as such offer the potential to predict transmission risk even in areas for which no sampling is available based on their environmental characteristics. Of course, still other factors could and should be incorporated into this view: access to constant potable water (eliminating the need for rain water catchments), urban and economic structure, population behavior, *etc.* This study, however, sets out to assess the overall feasibility of risk mapping for DF case occurrences—our conclusion is that effective risk maps can be developed based on such analyses of presence-only occurrence data.

## Figures and Tables

**Table 1.** Summary of Landsat bands used as input environmental datasets in development of ecological niche models.

Landsat bands	Band characteristics
1 (blue-green)	Useful for soil-vegetation differentiation.
2 (green)	Differentiates green reflectance from healthy vegetation
3 (red)	Detect chlorophyll absorption in vegetation
4 (near-infrared)	Detect near-infrared reflectance peaks in healthy green vegetation, and water-land interfaces
5 and 7 (mid-infrared)	Useful in characterizing vegetation and soil moisture.
6.1 (far-infrared)	Designed to assist in thermal mapping, and for soil moisture and vegetation studies

**Table 2.** Summary of model predictions and tests in this study. B = Bello (N = 113), M = Medellín (N = 611), I = Itagüí (N = 52). The commission error index is the proportion of the test region predicted present at a given threshold [28]. Proportions of correct predictions out of total numbers of test points are given, along with the associated cumulative binomial probabilities. The arrow (→) indicates the area being predicted. The “model” column shows the variables used to build the model.

		**Commission error index**	**Omission error**	**Probability**
**Within municipality**				
B: 1→2	no aspect, no slope, no band 6	0.57	0.89	9.33 × 10^−15^
B: 2→1	no aspect, no slope, no elevation, no band 6	0.59	0.90	7.77 × 10^−15^
B→M	no aspect, no slope, no band 6	0.49	0.62	3.40 × 10^−9^
B→I	no aspect, no slope, no band 6	0.46	0.81	1.40 × 10^−5^
**Between municipalities**				
BM→I	no aspect, no elevation	0.63	0.56	1.64 × 10^−3^
MI→B	no aspect, no slope	0.55	0.64	2.38 × 10^−5^
BI→M	Landsat only	0.45	0.68	3.12 × 10^−11^
